# How does autophagy impact neurological function?

**DOI:** 10.1177/10738584251324459

**Published:** 2025-03-13

**Authors:** Angeleen Fleming, Ana Lopez, Matea Rob, Sarayu Ramakrishna, So Jung Park, Xinyi Li, David C. Rubinsztein

**Affiliations:** 1 Department of Medical Genetics, University of Cambridge, Cambridge Institute for Medical Research, Cambridge, UK; 2 Department of Physiology, Development and Neuroscience, University of Cambridge, Cambridge, UK; 3 UK Dementia Research Institute, University of Cambridge, Cambridge Institute for Medical Research, Cambridge, UK

**Keywords:** autophagy, neuron, neural stem cell, aggregates, neuroinflammation, glia

## Abstract

Autophagies describe a set of processes in which cells degrade their cytoplasmic contents via various routes that terminate with the lysosome. In macroautophagy (the focus of this review, henceforth autophagy), cytoplasmic contents, including misfolded proteins, protein complexes, dysfunctional organelles, and various pathogens, are captured within double membranes called autophagosomes, which ultimately fuse with lysosomes, after which their contents are degraded. Autophagy is important in maintaining neuronal and glial function; consequently, disrupted autophagy is associated with various neurologic diseases. This review provides a broad perspective on the roles of autophagy in the CNS, highlighting recent literature that furthers our understanding of the multifaceted role of autophagy in maintaining a healthy nervous system.

## Introduction

**Figure 1.**
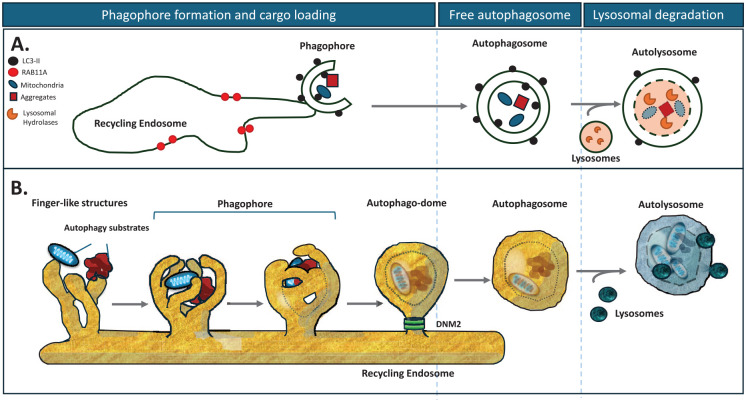
Schematic representation of macroautophagy. (**A**) In schematic representations of the process of (macro)autophagy, phagophores are typically represented as cup-shaped, double-membraned structures emerging from the recycling endosome. As the phagophore closes, it traps autophagic cargo, forming a double-membraned vesicle called the autophagosome. Autophagosomes then fuse with lysosomes to form autolysosomes, in which the contents are degraded by lysosomal enzymes. (**B**) Recent work has revealed that autophagosomes form from finger-like structures, which “grow” from the recycling endosome (rather than from a cup-shaped structure, as depicted in **A**). These finger-like projections surround a portion of cytoplasm and substrates, with the cargo still visible through the gaps between the fingers of these autophagosome precursors. The phagophore finally closes in an ESCRT-dependent manner but remains attached to the surface of the recycling endosome, forming a structure termed the “autophago-dome.” Dynamin 2 (DNM2) excises this closed structure from the recycling endosome, releasing the autophagosome such that it can move within the cytoplasm and traffic to the lysosomes for fusion and cargo degradation. ESCRT = endosomal sorting complexes required for transport.

Autophagies describe a set of processes whereby cells enable lysosomal degradation of cytoplasmic contents. Macroautophagy (the focus of this review, henceforth autophagy) captures cytoplasmic contents, including misfolded proteins, protein complexes, dysfunctional organelles, and various pathogens within double membranes called autophagosomes, which ultimately fuse with lysosomes, after which their contents are degraded (Fig. 1A). Chaperone-mediated autophagy uses the cytoplasmic HSC70 protein to bind to proteins with exposed pentapeptide motifs similar to KFERQ. The HSC70 binds to LAMP2A, which serves as a channel in the lysosome, which translocates the chaperone-mediated autophagy substrates into lysosomes, where they bind lysosomal HSC70. Microautophagy describes the entry of proteins into the endocytic-lysosomal system by invagination of endosomes or lysosomes (Nixon and Rubinsztein 2024).

Autophagosome formation involves the coordinated actions of a series of proteins with different functions. Many of these proteins are named ATG(n), where ATG is an abbreviation for autophagy-related. The defining event in autophagosome biogenesis is the ubiquitin-like conjugation of LC3 family members to phosphatidylethanolamine in nascent autophagosome membranes. The E3 ligase-like complex that mediates this event comprises the ATG5-ATG12 conjugate and ATG16L1 (Mizushima 2020). The location of this complex determines which membranes are conjugated by LC3-family proteins (Fujita and others 2008). ATG16L1 localization is regulated by its interaction with WIPI2 (Dooley and others 2014). WIPI2 conjugation to nascent autophagosome membranes is enabled by coincident binding to both the lipid phosphatidylinositol 3-phosphate (produced by the Beclin 1–containing VPS34 complex) and the recycling endosome protein RAB11A (Puri and others 2018). LC3-positive autophagosomes form as finger-like outgrowths from the RAB11A compartment. These capture cytoplasmic contents as the “fingers” close into a “fist” (Puri and others 2023) and the apertures are sealed by components of the endosomal sorting complexes required for transport (ESCRT) protein machinery (Takahashi and others 2018). This results in the formation of a short-lived intermediate—a closed autophagosome with its captured contents attached to the recycling endosome compartment. The autophagosomes are then released from this compartment by dynamin 2–dependent scission (Puri and others 2020) and are trafficked to and ultimately fuse with the lysosome, enabling the degradation of their contents (Fig. 1B).

Autophagy can be considered both a bulk process where cytoplasmic contents are “randomly” captured into autophagosomes and a selective process, termed selective autophagy, where substrates such as aggregate-prone proteins (aggrephagy), dysfunctional mitochondria (mitophagy), endoplasmic reticulum (ER) (ER-phagy), and peroxisomes (pexophagy), among many related processes, are selected for incorporation into autophagosomes by binding to bifunctional autophagy receptors. These autophagy receptors, exemplified by P62 (also known as SQSTM1), among a large set of such proteins, enable selective autophagy processes as they interact with their substrates (typically after they are ubiquitinated with K63 chains) and also bind to core autophagy machinery (generally LC3 family members) (reviewed in Nixon and Rubinsztein 2024).

In this review, we will reveal some of the different consequences of autophagy defects on various aspects of neuronal and glial function in health and disease. We will not specifically focus on different neurologic/neurodegenerative diseases and their connections with autophagy, as this has been considered elsewhere recently (Nixon and Rubinsztein 2024; Palmer and others 2024) but rather hope that this review may provide a broader perspective on the roles of autophagy in the CNS. Please note that we cannot be comprehensive about all the roles of autophagy in such a review but have instead focused on a selected set of processes from more recent literature that we believe are important. While we have divided the review into sections considering the roles of autophagy in normal physiology and CNS maintenance and autophagy and disease, these subdivisions are fluid as there is considerable overlap between the two.

## Autophagy in Normal Development and Physiology

### Autophagy in Development of the Nervous System

Autophagy plays an essential role in neurogenesis, both in the development of the embryonic nervous system and in adult neural stem cells (NSCs), which reside within the subgranular zone in the hippocampal dentate gyrus and the subventricular zone around the lateral ventricles. There is elevated expression of core autophagy genes in progenitor cells within the CNS during embryonic neurogenesis (Wu and others 2013), and silencing autophagy-regulating genes in the developing CNS results in defects in neuronal proliferation and differentiation (Fleming and Rubinsztein 2020). This is likely caused by aberrant Notch signaling since Notch1, a key regulator of stem cell differentiation, is degraded by autophagy (Wu and others 2016) (see “Autophagy and the Regulation of Cell Surface Proteins”). It is not surprising, therefore, that mutations in key autophagy genes result in childhood neurodevelopmental disorders (reviewed in Fleming and Rubinsztein 2020; Lienard and others 2024).

An additional emerging role for autophagy during neurogenesis is in the remodeling of intracellular organelles. Using the differentiation of human embryonic stem cells into induced neurons (iNeurons) as an in vitro model of neurogenesis, proteomics analysis of autophagy-null versus autophagy-competent differentiating iNeurons identified an accumulation of ER, Golgi, and mitochondria (Ordureau and others 2021). Within axons, the ER undergoes continuous remodeling for axon repair, neurotransmission, and the maintenance of axonal homeostasis (Yperman and Kuijpers 2023). ER remodeling is also essential for neurite growth, and recent evidence suggests that this is likely through selective autophagy, known as ER-phagy (Hoyer and others 2024), or through a secretory route that is dependent on autophagy machinery, termed secretory reticulophagy/ER-phagy (SERP) (Wojnacki and others 2020).

The role of autophagy in adult NSC maintenance is less clear. While enhancing autophagy through the expression of active Beclin-1 protects adult NSCs from exhaustion and promotes neurogenesis in *Becn1*^F121A/F121A^ mice (Wang and others 2022), conditional knockout (cKO) of key autophagy genes in adult neural stem cells has produced conflicting results. Retroviral Cre injections into dividing NSCs in the dentate gyrus of adult *Atg5*^flox/flox^ mice resulted in reduced survival of stem cell progenitors and delayed neuronal maturation (Xi and others 2016), whereas no effect on self-renewal or differentiation was observed in *Atg5, Atg7*, and *Atg16L1* cKO mice generated with the hGFAP:Cre driver (Wang and others 2016a). While the latter study did observe depleted adult neural stem cell progenitors and decreased neurogenesis in *FIP200* cKO mice (Wang and others 2016a), subsequent work has demonstrated that this role for FIP200 in adult NSC maintenance is mediated by its noncanonical (i.e., non-autophagy) functions. In the NSCs of knock-in mice expressing a mutated form of FIP200 (FIP200-4A) that cannot interact with ATG13, TBK1 activation and P62/SQSTM1 phosphorylation was observed (Liu and others 2021). Since previous work from the same group had elucidated that aggregation of P62/SQSTM1 and its role in regulating superoxide dismutase accounted for the differences observed between *FIP200* and *Atg5, Atg7*, and *Atg16L1* cKO mice, the apparent discrepancy can be explained by the non-autophagy functions of FIP200. In addition, this group demonstrated that P62/SQSTM1 aggregation and increased chemokine expression in NSCs resulted in microglial activation and infiltration into the stem cell niche, resulting in a non-cell-autonomous effect on NSC maintenance (Wang and others 2017).

### Autophagy and the Maintenance of Neuronal Function

Autophagosomes form in the presynaptic compartment and distal portion of axons and in the presynaptic compartment, and they undergo retrograde transport along the axon to the cell body, where they fuse with lysosomes (reviewed in Nixon and Rubinsztein 2024). However, the physiologic substrates of these autophagosomes have only recently been elucidated. In *Atg5* cKO mice, loss of neuronal autophagy caused a cell-autonomous elevation of presynaptic neurotransmission without changes in the number or density of synapses or presynaptic vesicle numbers, localization, or levels of key synaptic proteins. The absence of ER-phagy resulted in an axonal accumulation of tubular ER, leading to elevated calcium release from ER stores via ryanodine receptors accumulated in axons and at presynaptic sites (Kuijpers and others 2021).

These findings are supported by quantitative proteomics of cKO of *Atg5* in either excitatory or inhibitory neurons, which confirmed that loss of autophagy does not affect the levels of synaptic vesicle proteins and found highly upregulated levels of ER-phagy receptors TEX264 and SEC62 in inhibitory neurons (Overhoff and others 2022). The main finding of this study, however, was that regulatory subunits of protein kinase A (PKA) holoenzyme R1α (PRKAR1A) and R1β (PRKAR1B) were the most significantly upregulated proteins in both neuronal types. Autophagy-dependent degradation of these enzymes was shown to be essential in regulating synaptic levels of Ca^2+^ permeable GLUR1-containing AMPA receptors (Overhoff and others 2022). Since an increase in surface localization of synaptic AMPA receptors triggers abnormal synchronous activity and can lead to epileptic seizures (reviewed in Guo and Ma 2021), this would account for the recurrent seizures in mice lacking ATG5 in excitatory forebrain neurons.

Within the postsynaptic terminal, stimulation of synaptic activity dampens autophagic vesicle motility, whereas silencing synaptic activity induces motility, suggesting a mechanism whereby activity can regulate the postsynaptic proteome (Kulkarni and others 2021). Evidence for the physiologic relevance of this comes from a study demonstrating that induction of long-term depression in mice initiates autophagy in dendrites. Activation of NMDA receptors or metabotropic glutamate receptors initiates autophagy in the postsynaptic dendrites in mice. This, along with endocytosis, resulted in the removal and degradation of AMPA receptor subunits from the membrane and in *Atg5* cKO mice, resulting in abolition of LTD (Kallergi and others 2022).

### Autophagy and the Regulation of Cell Surface Proteins

**Figure 2.**
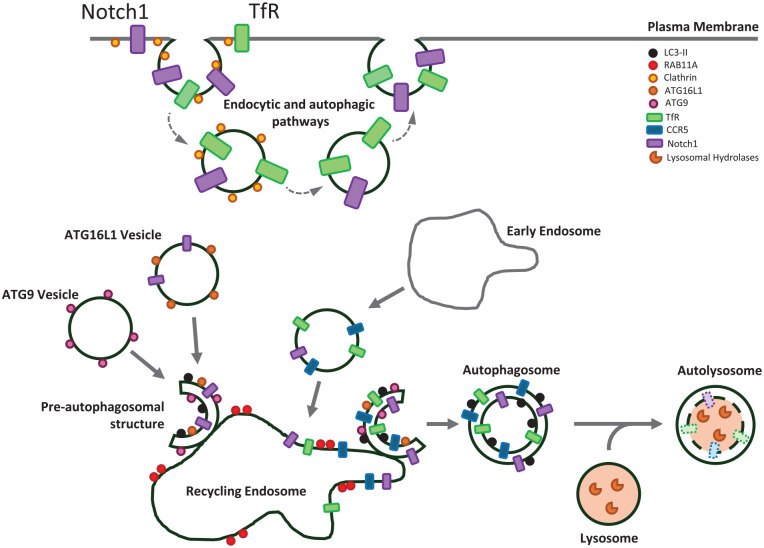
Plasma membrane receptors enter autophagosomes via the recycling endosome. Plasma membrane receptors can be incorporated into autophagosomes via the recycling endosome. The process begins at the plasma membrane, where receptors are embedded in the phospholipid bilayer. CCR5 and transferrin receptor (TfR) are internalized via clathrin-coated pits, traversing early endosomes, before arriving at recycling endosomes. In addition to this canonical endocytic pathway, Notch1 can enter ATG16L1-positive vesicles directly from the plasma membrane. ATG16L1 vesicles are transported from the plasma membrane to recycling endosomes, bypassing the early endosomal compartment. The recycling endosome, marked by RAB11A, serves as a meeting point for ATG16L1 and ATG9, an essential step in autophagy initiation. Autophagosomes form from the recycling endosome membranes, along with the internalized receptors. In the final stage, a mature autophagosome, with its characteristic double membrane, now contains the internalized receptors. The mature autophagosome fuses with a lysosome, forming an autolysosome. The fusion event allows the lysosomal hydrolases to access the autophagosome contents, including the plasma membrane receptors that are embedded in the inner autophagosome membrane. These hydrolases then break down the inner autophagosome membrane and its associated proteins that were trafficked from the plasma membrane, effectively completing the process of receptor downregulation.

Receptor endocytosis is a critical process that allows cell surface receptors to internalize extracellular molecules, initiating intracellular signaling pathways or enabling uptake of nutrients. Upon internalization, these receptors can either be degraded or recycled back to the plasma membrane, thereby modulating receptor availability and signal transduction (Cullen and Steinberg 2018). Autophagy regulates the degradation of plasma membrane receptors, impacting physiologic processes and disease mechanisms. Two examples are the transferrin and Notch1 receptors (Fig. 2).

Recycling endosomes play a central role in protein sorting and recycling, and they also serve as sites for autophagosome assembly (Puri and others 2018). This dual function facilitates the targeting of cell surface receptors, such as the transferrin receptor (TfR), toward autophagic degradation via recycling endosomes. TfR is critical for iron uptake into CNS cells. Under starvation conditions, internalized TfR localizes to recycling endosomes marked by RAB11A and colocalizes with autophagic markers like LC3 and ATG16L1 (Puri and others 2018). Electron microscopy has shown that erstwhile extracellular epitopes of the TfR are labeled with immunogold between the inner and outer membranes of autophagosomes, indicating a noncanonical degradation process via autophagy, where a transmembrane receptor integrates into the recycling endosome/autophagosome membrane. In this way, autophagy regulates TfR levels, which, in turn, impact cellular iron homeostasis.

Autophagy also plays a crucial role in modulating the expression of Notch1, a plasma membrane receptor regulating stem cell differentiation and embryonic development. Notch1 levels are regulated by autophagy through two distinct pathways: the conventional endocytic route and a novel endocytosis-independent mechanism where Notch1 is internalized directly from the cell surface into ATG16L1-positive pre-autophagosomal structures (Wu and others 2016). ATG16L1 and ATG9 vesicle fusion at RAB11A-positive recycling endosomes is crucial for autophagosome formation (Puri and others 2013), suggesting that, like TfR, Notch1 may also enter the autophagosome through this cellular compartment. In ATG16L1 hypomorphic mice, reduced autophagy increases Notch1 signaling, inhibiting neuronal differentiation (Wu and others 2016), further underscoring the importance of autophagic regulation of cell surface receptors in neuronal homeostasis.

### Autophagy and Maintenance of Genomic Integrity

Besides its role in the degradation of cytoplasmic contents, autophagy also contributes to maintaining genome integrity (Hewitt and Korolchuk 2017). If DNA damage is increased, this leads to neurodegeneration, for example, in neurodevelopmental disorders, senescence (“Senescence” section), and neuroinflammation (“Inflammation” section) (Welch and Tsai 2022). The connection between autophagy and genomic stability was first suggested when monoallelic loss of Beclin-1, a key autophagic protein, was observed to result in increased DNA damage in breast cancer cells (Karantza-Wadsworth and others 2007). More recent studies have further highlighted the direct involvement of autophagy in DNA damage repair mechanisms. Recently, Topoisomerase 1 cleavage complex was found to be exported from the nucleus and then selectively degraded through autophagy, and the process was orchestrated by TEX264 (Lascaux and others 2024). Additionally, autophagic proteins have been found to participate directly in the regulation of DNA repair processes. UV radiation resistance-associated gene (UVRAG), which forms a distinct autophagic complex with Beclin-1 and VPS34, was also found to directly interact with DNA-PK complexes, thereby facilitating non-homologous end joining (NHEJ) repair (Zhao and others 2012). Moreover, the core autophagy protein Beclin-1 has been reported to actively translocate into the nucleus, where it confers protection against radiation-induced DNA damage (Xu and others 2017).

Interestingly, the autophagic receptor protein P62/SQSTM1 has been identified to shuttle into the nucleus, where it interacts with the E3 ligase RNF168, inhibiting its ubiquitination activity. RNF168 plays a pivotal role in chromatin ubiquitination and the recruitment of DNA damage response proteins following genotoxic stress. In cases of autophagy impairment, the accumulation of nuclear P62/SQSTM1 enhances its interaction with RNF168, potentially leading to increased genomic instability (Wang and others 2016b).

Furthermore, autophagy also modulates the levels of essential DNA repair proteins, thereby influencing genome stability. CHK1 (checkpoint kinase 1), a key regulator of homologous recombination repair—a relatively error-free mechanism that uses an endogenous template for DNA repair—is one such example. CHK1 turnover is typically mediated by the ubiquitin-proteasome pathway. When autophagy is compromised, proteostasis is maintained through heightened proteasomal activity. This increases CHK1 depletion and shifts DNA repair mechanisms toward the error-prone NHEJ pathway, resulting in accumulation of genome instability in the long term (Liu and others 2015).

Beyond the abovementioned pathways, autophagy may also play a protective role in mitigating DNA damage by reducing reactive oxygen species (ROS) levels by eliminating damaged organelles such as mitochondria via mitophagy (Kurihara and others 2012). The identification of these novel roles for autophagy in maintaining genome integrity could potentially guide new therapeutic opportunities.

## Autophagy and Disease-Associated Processes

### Aggrephagy

Autophagy plays a well-established role in degrading intracytoplasmic aggregate-prone proteins, like mutant huntingtin, tau, and alpha-synuclein, which cause Huntington’s disease, various dementias, and Parkinson’s disease, respectively (Williams and others 2006). Aggrephagy is the selective autophagy of cytoplasmic aggregated/aggregate-prone proteins mediated via well-characterized aggrephagy receptors such as P62/SQSTM1, NBR1, TAX1BP1, and TOLLIP (Vargas and others 2023). Each aggrephagy receptor has different binding partners and cargo selectivity (see Table 1), and mutations in several aggrephagy receptors are associated with amyotrophic lateral sclerosis. Autophagy appears to target amorphous aggregates rather than more mature and larger fibrils, suggesting that the degradation process targets early intermediates in the aggregation process (Zhao and others 2024). As many neurodegenerative disease-causing proteins, like mutant huntingtin and alpha-synuclein, are autophagy substrates and also inhibit autophagy (Ashkenazi and others 2017; Winslow and others 2010), this may lead to a positive-feedback cycle, once cells cannot buffer the accumulation of such proteins. Upregulating the autophagic degradation of such proteins has been successful in ameliorating signs of neurodegeneration in animal models of such diseases both by boosting bulk autophagy and by creating artificial autophagy receptors, enabling the degradation of such substrates via selective autophagy (Fleming and others 2022).

**Table 1.** Cargo Selectivity of Aggrephagy Receptors and Mutations Associated with Neurodegenerative Disease.

Aggrephagy receptor	Binding partners	Role in clearance of aggregated proteins/Association with disease	Reference
P62/SQSTM1	NBR1, WDR81, ALFY, FIP200	Degradation of aggregated MAPT/Tau	Xu and others 2019; Ono and others 2022
		Clearance of neuron-released α-synuclein by microglial P62/SQSTM1	Choi and others 2020
		L341V mutation of SQSTM1 identified in ALS patients. L341V leads to defective recognition of LC3B and limits the SQSTM1 recruitment to the phagophore	Goode and others 2016
		ALS-FTLD-linked mutation G427R of SQSTM1 reduces Nrf2-targeted gene expression and increases TARDBP/TDP-43 associated stress granule formation under oxidative stress	Deng and others 2020
NBR1	P62, TAXBP1, FIP200	NBR1 detected in Lewy bodies and glial cytoplasmic inclusions in alpha-synucleopathies	Odagiri and others 2012
TAXBP1	NBR1, FIP200	Clearance of stress-induced aggregates, polyQ-HTT and ALS-associated protein TDP43	Sarraf and others 2020
OPTN	Rab8, HACE1, P62	Clearance of stress-induced protein aggregates and Parkin-dependent clearance of damaged mitochondria	Slowicka and others 2016
		OPTN mutations reported in ALS patients - a homozygous deletion of exon 5, a homozygous Q398X nonsense mutation and a heterozygous E478G missense mutation within its ubiquitin-binding domain.	Maruyama and others 2010
		Q398X mutation causes mis-localization of TDP43 leading to degeneration of motor neurons	Kurashige and others 2021
		E478G mutation increases inflammation and neuronal cell death	Liu and others 2018
CCDC50		Clearance of stress-induced polyubiquitinated protein aggregates	Ye and others 2024
TOLLIP	Parkin	Protection of cells against the toxicity of polyQ-expanded HTT by facilitating their transport to late endosome	Shimizu and others 2014; Oguro and others 2011
		Reduced TOLLIP expression in human brain samples of aged and Alzheimer's disease individuals as compared to young individuals	Cribbs and others 2012
CCT2		Clearance of solid aggregates such as polyQ-HTT in a ubiquitin-independent manner	Ma and others 2022

### Autophagy and Cell Death Pathways

Neuronal cell death is a major pathologic hallmark of neurodegenerative diseases and is also important for development. Cell death pathways have crosstalk with autophagy, which may impact various neuropathologic conditions (Moujalled and others 2021).

#### Apoptosis

Apoptosis is a caspase-mediated programmed cell death manifesting with cell shrinkage, membrane blebbing, and chromatin condensation. The BCL-2 family proteins play critical roles in apoptosis and autophagy. The anti-apoptotic proteins BCL-2 and BCL-XL inhibit autophagy by binding to Beclin-1, but replacing these with pro-apoptotic BH3-only proteins reverses this effect (Levine and others 2008; Luo and Rubinsztein 2010). BAX activation by oxidative stress is regulated by the p53 upregulated modulator of apoptosis (PUMA), causing selective mitochondrial removal by autophagy and neuronal apoptosis (Steckley and others 2007; Yee and others 2009). Since autophagy keeps PUMA levels low, this protects neurons from Aβ-induced apoptosis (Saha and others 2021; Thorburn and others 2014). Increased ROS generation by the neurotoxin 6-hydroxydopamine (6-OHDA) causes hyperactivation of autophagic flux, contributing to subsequent caspase-3-dependent apoptosis in neuronal cells and mouse cortical neurons. Indeed, apoptosis triggered by 6-OHDA is attenuated by pharmacologic inhibition of autophagy with 3-methyladenine or by genetic deletion of the autophagy-related gene *Atg5* (Chung and others 2018). Taken together, growing evidence suggests the importance of crosstalk between autophagy and apoptosis in the neuronal system.

#### Necrosis

Necrosis exhibits morphologic features characterized by plasma membrane rupture and cytoplasmic organelle swelling. Necrosis is regarded as an uncontrolled form of cell death. A programmed form of necrosis known as necroptosis is regulated by receptor-interacting protein kinase 1 (RIPK1), receptor-interacting protein kinase 3 (RIPK3), and mixed lineage kinase domain-like (MLKL) (Cui and others 2021). Inhibition of necroptosis by the RIPK1 inhibitor necrostatin-1 has a neuroprotective effect in glutamate-induced excitotoxic death of cortical neurons, in a Huntington’s disease mouse model, as well as in mouse primary neurons during hypoxia and ischemic brain damage (Mitroshina and others 2022; Zhu and others 2011). Furthermore, autophagy activation by mTOR inhibition reduces zVAD-induced necrotic cell death (Wu and others 2008). The recruitment of RIPK1 to P62/SQSTM1 on autophagosomes regulates necrosome assembly for tumor necrosis factor α (TNFα)–related apoptosis-inducing ligand (TRAIL)–induced necroptosis. Inhibition of this recruitment shifts the process toward apoptosis (Goodall and others 2016). Since p53 accumulation is implicated in neuronal necrosis, the upregulation of small chaperones and autophagy can prevent neuronal necrosis by promoting p53 degradation in *Drosophila*, rat, and mammalian cells (Lei and others 2017).

#### Ferroptosis

Ferroptosis is an iron-dependent form of cell death characterized by iron overload and subsequent lipid peroxidation, linking brain iron accumulation to neuronal death. Ferroptosis inhibitors have been shown to effectively protect against neuronal death caused by ischemic injury in mouse brains and in models of neurodegenerative brain disorders (Stockwell and others 2017).

Autophagy-associated ferroptosis can be facilitated by degradation of ferritin through a selective autophagy process, called ferritinophagy, mediated by the autophagy receptor nuclear receptor activator 4 (NCOA4), leading to the release of free iron from ferritin in the lysosome (Mancias and others 2014). The liberated free iron facilitates the lipid peroxidiation, leading to ferroptosis. Loss-of-function mutations in the WD repeat domain 45 (*WDR45*) gene, which encodes WIPI4, lead to β-propeller protein-associated neurodegeneration (BPAN), a rare X-linked dominant disease characterized by neurodegeneration and brain iron accumulation. Although WIPI4 is involved in autophagy, its inhibition triggers ferroptosis by enhancing the transfer of phosphatidylserine from the ER to mitochondria, independent of autophagy (Zhu and others 2024). This phosphatidylserine (PS) is converted to phosphatidylethanolamine (PE) in mitochondria, which is a major lipid modified by peroxidation in ferroptosis, and inhibiting this PS-to-PE conversion blocks ferroptosis caused by WIPI4 loss (Zhu and others 2024).

#### Synthetic lethality

Synthetic lethality is a type of genetic interaction in which the perturbation of two genes leads to unexpected cell death, whereas the disruption of each gene alone does not cause cell death (Costanzo and others 2019; Ooi and others 2006). Synthetic lethal interactions of core autophagy genes with nuclear pore complex components or proteasome subunits, as seen in yeast, cause synergistic viability changes akin to synthetic fitness loss. This situation arises since autophagy-deficient cells route cytoplasmic (autophagy) substrates to the nucleus for proteosomal degradation. These negative genetic interactions may be relevant in Huntington’s disease, where synergistic vulnerabilities occur due to modest autophagy defects and impaired nuclear import (Park and others 2024).

### Senescence

Cellular senescence is a nonproliferative state characterized by cell cycle arrest or the senescence-associated secretory phenotype that occurs after stress, culminating in a distinctive change in cell state. Impairments of autophagy predispose cells to senescence as they accumulate the transcription factor GATA-4 (Kang and others 2015). Age-related autophagy alterations have been observed in the brains of senescence-accelerated mice, along with learning and memory deficits (Ma and others 2011). In normal brain aging, autophagy genes are transcriptionally downregulated with age in both *Drosophila* and postmortem human brains (Hansen and others 2008; Lipinski and others 2010). Vinexin, a regulator of the actin cytoskeleton, negatively controls autophagosome biogenesis through YAP1/TAZ transcriptional activity. Increased vinexin expression is correlated with autophagic decline during normal brain aging in mice and humans (Park and others 2022). Interestingly, autophagy compromise resulting from mTORC1 activation is seen in cells from patients with the rare autosomal-dominant premature aging disorder Hutchinson–Gilford progeria syndrome (Son and others 2024).

### Inflammation

Chronic neuroinflammation is a hallmark of age-related neurodegenerative diseases (Zhang and others 2023b). Microglia, the resident immune cells of the CNS, maintain neuronal health by clearing extracellular protein aggregates and orchestrating immune responses. In neurodegenerative conditions, microglial hyperactivation leads to increased secretion of proinflammatory cytokines such as TNFα, IL-1β, and IL-6 (Hickman and others 2018). The persistent elevation of these cytokines can have detrimental effects on neuronal survival and function. Notably, activated microglia have been shown to inhibit neuronal autophagy, which is detrimental to neuronal health (Festa and others 2023).

When microglia are activated, they secrete chemokines (CCL3, CCL4, and CCL5) that inhibit neuronal autophagy. The chemokines bind and activate their receptor, the G-protein coupled receptor, CCR5. Neuronal CCR5 activation stimulates mTORC1 activity, which inhibits autophagy, causing an elevation of aggregate-prone proteins. Furthermore, CCR5 is an autophagy substrate that is degraded via the same route as TfR, described above. Since CCR5 activation impairs autophagy, which, in turn, compromises effective CCR5 degradation, this results in a self-sustaining cycle (Festa and others 2023) and illustrates how the reciprocal regulation between plasma membrane receptors and autophagy can drive disease progression.

Autophagy plays a crucial role in regulating microglial activity and inflammation. The NLRP3 inflammasome, which drives IL-1β and IL-18 maturation, has been identified as an autophagy substrate (Houtman and others 2019), directly connecting autophagy to inflammation regulation. Beclin-1 haploinsufficient microglia exhibit increased NLRP3 activation (Houtman and others 2019), while microglia-specific deletion of *Atg5* results in increased neuroinflammation and Parkinson’s disease–like symptoms in mice (Cheng and others 2020). Additionally, deletion of *Atg7* disrupts lipid metabolism in microglia, exacerbating NLRP3 activation and promoting tau pathology (Xu and others 2021). These findings suggest that autophagy not only is crucial for maintaining microglial function and buffering inflammatory processes but, through these routes, may also attenuate the progression of neurodegenerative diseases.

Autophagy intersects with another key regulator of inflammation, RIPK1. RIPK1 regulates TNF-induced cell death, balancing between apoptosis and necroptosis (see “Autophasy and Cell Death Pathways”). This regulation is intricately linked to autophagy; for instance, phosphorylation of RIPK1 by ULK1 reduces TNF-induced necroptosis, while knockdown of ULK1 enhances necroptosis (Wu and others 2020). Furthermore, RIPK1 negatively regulates basal autophagy through ERK-dependent phosphorylation of TFEB (Yonekawa and others 2015), indicating a complex interplay between these signaling pathways. In an Alzheimer’s disease mouse model, RIPK1 inhibition reduced amyloid burden in vivo, suppressed inflammatory cytokine secretion, and promoted microglial degradation of Aβ in vitro (Ofengeim and others 2017), suggesting that targeting inflammatory mediators could be a viable treatment strategy for neurodegeneration.

Autophagy is also vital for eliminating intracellular pathogens. In macrophages, myeloid cells function similarly to microglia, and the activation of pathogen-recognizing receptors, such as TLR7 (Delgado and others 2008), TLR2 (Chang and others 2013), and TLR4 (Lee and others 2019), promotes autophagy. Notably, TLR4 activation leads to NF-κB pathway activation and the expression of TNFα, IL-1β, and IL-6, key mediators of the inflammatory response. Interestingly, while TLR4 activation in macrophages induces autophagy, it suppresses autophagic flux in microglia by inhibiting the transcription factor FOXO3 (Lee and others 2019). This highlights a key distinction in the inflammatory regulation of autophagy between these two cell types and emphasizes the need for cell-specific analysis when studying autophagy in neuroinflammatory conditions.

Mitophagy is thought to act as a buffer against neuroinflammation. One major mitophagy pathway is mediated by the kinase PINK1 and the E3 ubiquitin ligase parkin. Compromise of this pathway causes activation of the cGAS-STING pathway (presumably via mitochondrial DNA), which causes a type I interferon inflammatory response (Moehlman and others 2023; Sliter and others 2018). This may be relevant in forms of Parkinson’s disease, especially those caused by recessive mutations in PINK1 or Parkin (Borsche and others 2020).

### Role of Autophagy in Oligodendrocytes

Oligodendrocytes are the exclusive myelin-producing cells in the CNS, and autophagy is important for their development, survival, and myelination. Autophagosome formation, autophagic flux, and expression of autophagy proteins increase during oligodendrocyte lineage development in vivo (Bankston and others 2019; Zhang and others 2023a). Different mechanisms are suggested for the role of autophagy in regulating myelination. Oligodendrocyte precursor cell (OPC)–specific deletion of *Atg5* in mice resulted in increased apoptosis of OPCs and reduction of mature oligodendrocytes, ultimately causing myelin deficiency (Bankston and others 2019), primarily in the corpus callosum. Conversely, an alternative model suggested decreased apoptosis of preoligodendrocytes in *Atg5* and *Atg7* cKO mice, leading to ectopic oligodendrocytes and myelination in the cerebellum, hippocampus, and cortex (Zhang and others 2023a). This shows a cell-autonomous role of autophagy in controlling oligodendrocyte number and the spatiotemporal specificity of myelination.

Mature oligodendrocytes rely on autophagy for regulating the turnover of myelin sheath proteins and hence maintaining functional neuronal circuitry (Aber and others 2022). Conditional depletion of *Atg7* in oligodendrocytes causes an age-dependent accumulation of myelin, increases myelin sheath thickness, and causes structural abnormalities, predominantly in the corpus callosum and optic nerve (Aber and others 2022). The myelin sheath abnormalities correlated with the tremors and motor deficits and preceded the neuronal loss in the aged *Atg7* cKO mice (Aber and others 2022). Selective autophagy of myelin binding protein (MBP), a cytosolic component of myelin sheath, occurs in a manner dependent on the selective autophagy receptors P62 and Alfy, whereby autophagosomes containing MBP form amphisomes (autophagosome-endosome fusions) with the endocytosed components of the myelin membrane, which are then targeted for lysosomal degradation (Aber and others 2022). Thus, autophagy coordinates the efficient degradation of both cytosolic and membranous components of myelin.

White matter in the brain is composed of oligodendrocytes and myelinated axons, and white matter injury is characterized by the loss of axonal integrity and demyelination. Although the functional role of autophagy in spinal cord injury is well reported in neurons and astrocytes, it is relatively less studied in oligodendrocytes. Chronic cerebral hypoperfusion has been used in rat models to induce white matter injury and results in an initial induction of autophagy in the white matter between three and seven days of injury. However, autophagy is suppressed by 14 days along with lysosomal dysfunction, resulting in the death of the mature oligodendrocytes (Wang and others 2023). Genetic deletion of *Atg5* in the OPCs or oligodendrocytes reduces cell viability and impairs the recovery from contusive spinal cord injury (Saraswat Ohri and others 2018). While pharmacologic inhibition of autophagy using drugs like KU55933 and verteporfin affected the oligodendrocyte myelination in vitro (Bankston and others 2019), autophagy activators such as rapamycin did not improve the locomotor recovery after injury (Saraswat Ohri and others 2018).

### Secretory Autophagy

**Figure 3.**
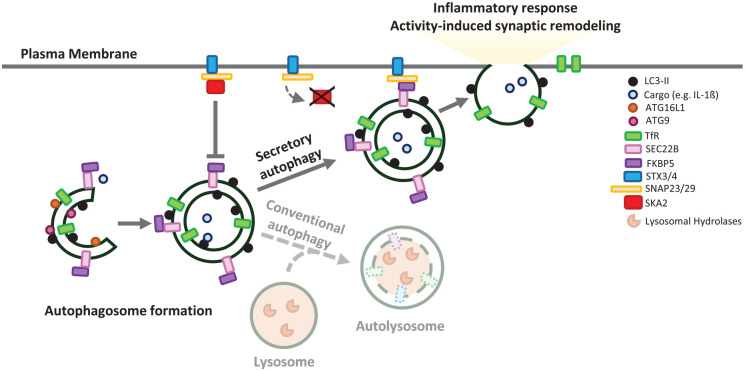
Secretory autophagy. Secretory autophagy is an alternative route to conventional autophagy in which autophagosomes fuse to the plasma membrane. This process is facilitated by the formation of a SNAP receptor (SNARE) complex between SEC22B and FKBP5 on the autophagosome and SNAP23, SNAP29, and the syntaxins STX3 and STX4 at the plasma membrane. The interaction of these SNAREs facilitates the fusion of the autophagic vesicle with the plasma membrane, releasing its cargo to the extracellular space. SCA2 prevents the formation of the SNARE-protein complex during vesicle–plasma membrane fusion, acting as a negative regulator of secretory autophagy. Secretory autophagy has been associated with remodeling of the synaptic terminal during neuronal activation and the release of proinflammatory cytokines such as IL-1β.

While the conventional route for autophagosome degradation is via fusion with lysosomes, an alternative fate for these vesicles is the secretory autophagy pathway (SA). SA shares the early steps of autophagosome formation with macroautophagy. However, during SA, formed autophagosomes fuse directly to the plasma membrane or multivesicular bodies (MVBs) to form amphisomes. These ultimately fuse with the cell membrane, releasing their cargo into the extracellular space (Fig. 3). This form of unconventional secretion can be triggered upon ER stress, starvation, lysosomal impairment, the unfolded protein response, or defects in the intracellular trafficking machinery (Li and others 2024). SA exports a range of cytoplasmic substrates from cytokines to organelles, contributing to cellular homeostasis and cell communication regulation, and hence is relevant in human diseases ranging from cancer to neurodegeneration (Li and others 2024).

SA is orchestrated by SNAP receptors (SNAREs) and RAB proteins, including SNAP29, SEC22B, RAB8A, RAB11A, and RAB27A (Li and others 2024). A new SA component has been recently identified, SK2A, which regulates neuroinflammation in the mammalian brain (Hartmann and others 2024). SKA2 inhibits the formation of the SNARE-protein complex during the docking of autophagosomes carrying IL-1β with the plasma membrane. Knockdown of SK2A in mice causes increased SA-dependent IL-1β release, which, in turn, promotes inflammasome activation, leading to cognitive impairment. Since the levels of SK2A are reduced in postmortem brains of patients with Alzheimer’s disease, one potential mechanism for elevated neuroinflammation may be via increased SA.

In addition, SA may play a key role in the progression of other neurogenerative diseases, like Parkinson’s disease, promoting α-synuclein secretion to the extracellular compartment. A recent study suggests that increased neuronal activity mediates α-synuclein secretion through SA in a Ca^2+^-dependent manner in primary cortical neurons stimulated by the addition of glutamate or by depolarization (Nakamura and others 2024).

On the other hand, maintenance of SA is essential for normal neuronal activity and plasticity (Chang and others 2024). In *Drosophila*, synapse development was shown to rely on canonical degradative autophagy, whereas the remodeling of synapses during neuronal activity depends on SA. An RNA interference (RNAi)–based genetic screen covering 398 *Drosophila* lines for unique orthologs of human disease-associated genes suggested that genes associated with neurodegenerative and mental disorders have a more disruptive effect on activity-induced synaptic remodeling than on synapse development, and this effect is SA dependent.

Although some cargo specificity has been described for secretory autophagosomes (such as IL-1β or viruses), less is known about others SA cargoes such as aggregation-prone proteins. Since both macroautophagy and SA rely on the same steps in autophagosome biogenesis, and disruption of these events occurs in many neurodegenerative diseases, a more detailed understanding of the mechanisms controlling these processes and how they overlap is required.

## Concluding Remarks

**Figure 4.**
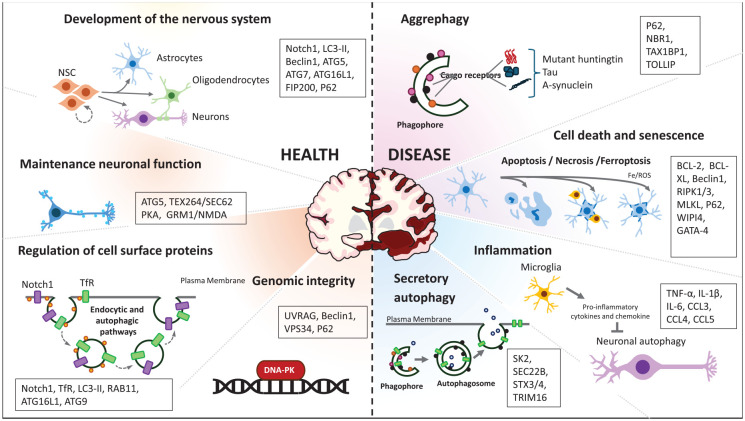
The roles of autophagy in the central nervous system. Autophagy is crucial for the development and maintenance of a healthy CNS, with defects in this process linked to neurodegenerative diseases. It participates in critical functions such as neurogenesis, neuronal proliferation, and differentiation during development, as well as sustaining the pool of neural stem cells (NSCs), astrocytes, oligodendrocytes, and neurons. Autophagy is also vital for preserving neuronal plasticity and neurotransmission by regulating the availability of signaling receptors at the plasma membrane, thus supporting neuronal function. Furthermore, autophagy plays an essential role in maintaining genomic stability by interacting with DNA repair mechanisms. The accumulation of aggregated or aggregation-prone proteins is a hallmark of neurodegenerative diseases, with selective clearance of these aggregates mediated by aggrephagy. Neuronal loss and cell cycle arrest are common in these diseases, where neurons undergo different forms of cell death, such as apoptosis, necrosis, or ferroptosis, all of which are linked to autophagy. Chronic inflammation due to microglial hyperactivation triggers the release of proinflammatory cytokines and chemokines that inhibit neuronal autophagy. Additionally, the fusion of autophagosomes with the plasma membrane during secretory autophagy has been associated with inflammasome activation and the secretion of IL-1β. The key proteins discussed in this review are noted in text boxes.

Autophagy buffers diverse processes in the CNS. Hence, even a modest compromise in autophagy may have a large global effect (summarized in Fig. 4). This will be due to cumulative dysfunction in a range of different systems impacting neuronal functions and homeostasis. Furthermore, the potential for positive feedback loops, as exemplified by microglial chemokine release triggered by the presence of neurotoxic neuronal proteins impairing neuronal autophagy, suggests that CNS autophagy needs to be properly maintained for normal health, function, and development. On the other hand, in diseases where there is some impairment in the autophagy-lysosome process (Nixon and Rubinsztein 2024), it is striking that function can be maintained for some time, suggesting that we need to understand more about compensatory mechanisms influencing protein degradation (ubiquitin proteasome system), protein folding (chaperones), and secretion and their interplay with autophagies.
